# Supplementary material for: The Influence of Smoking Status on Exhaled Breath Profiles in Asthma and COPD Patients
Source: Molecules. 2021 Mar 4;26(5):1357. doi: 10.3390/molecules26051357 (PMC7961431; doi:10.3390/molecules26051357)
Supplement: Supplementary file 1 [file molecules-26-01357-s001.pdf]

Supporting Information

The Influence of Smoking Status on Exhaled Breath Profiles in Asthma and COPD Patients

Stefania Principe [1,2], Job J.M.H. van Bragt [1], Cristina Longo [1], Rianne de Vries [1,3], Peter J. Sterk [1], Nicola Scichilone [2], Susanne J.H. Vijverberg [1], Anke H. Maitland-van der Zee [1,*]

[1] Department of Respiratory Medicine, Amsterdam UMC, University of Amsterdam, 1105 AZ Amsterdam, The Netherlands
[2] Dipartimento Universitario di Promozione della Salute, Materno Infantile, University of Palermo, Medicina Interna e Specialistica di Eccellenza "G. D'Alessandro"(PROMISE) c/o Pneumologia; AOUP "Policlinico Paolo Giaccone", 90127 Palermo, Italy
[3] Breathomix b.v., Leiden, 2333, Zuid Holland, The Netherlands

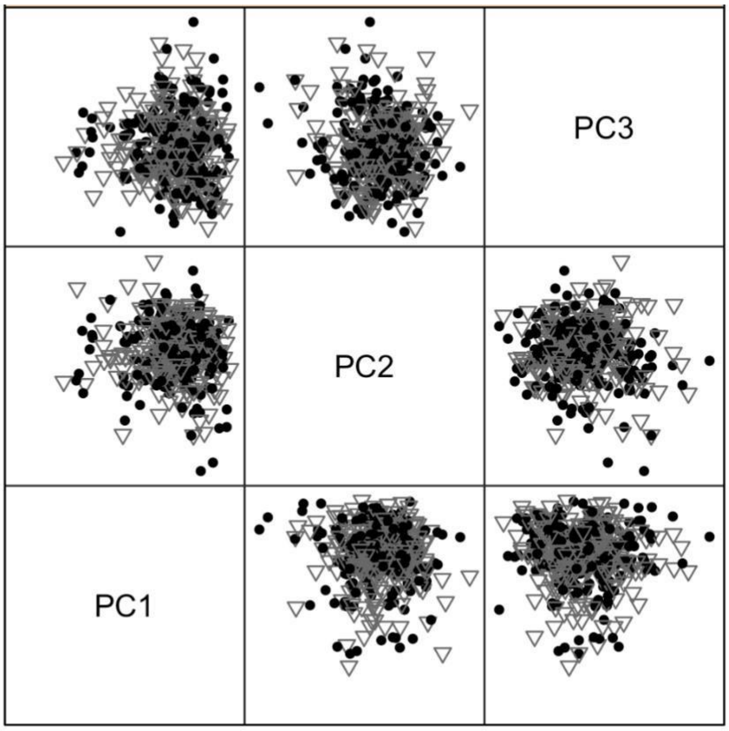

Figure S1. Scatter plot matrices, that shows the correlation between each Principal Components (PCs) in a population of ever or non-smokers in the overall dataset (n = 896): dotplots are representing never smokers and triangles ever smokers.

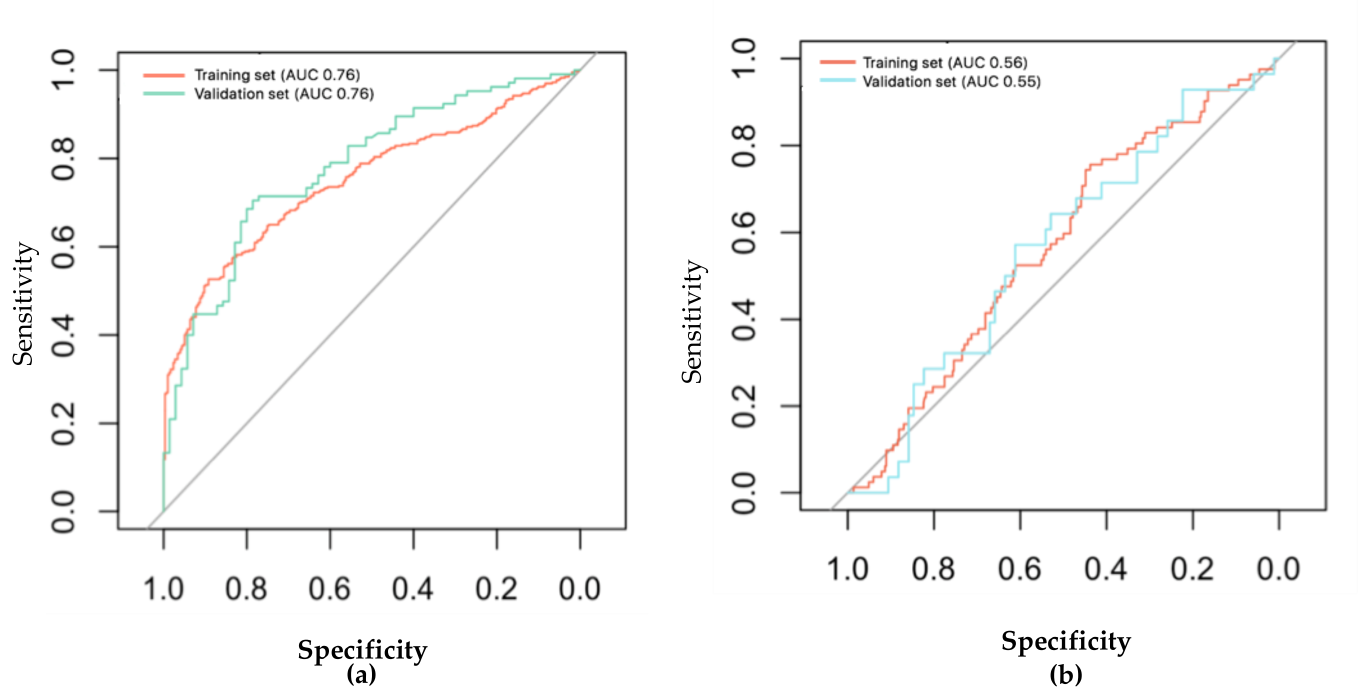

(a) **(b)**

Figure S2. ROC analyses showing the accuracy of the linear discriminant model based on principal component reduction in the training set and the independent validation set according to pack/years (less than 5 pack/years, more than 5 pack/years) for (a) asthma and COPD group: (Control: < 5 pack/years; Case: > 5 pack/years): Training set: Case = 397; Control = 297; 95% CI: 0.72–0.78; AUC: 0.76. Validation set: Case = 105; Control = 72; 95% CI: 0.71–0.84; AUC: 0.76, and (b) for healthy control group (Control: < 5 pack/years; Case: > 5 pack/years): Training set: AUC: 0.56 (95% CI: 0.50–0.61); Validation set: AUC: 0.55 (95%CI: 0.58–0.74).

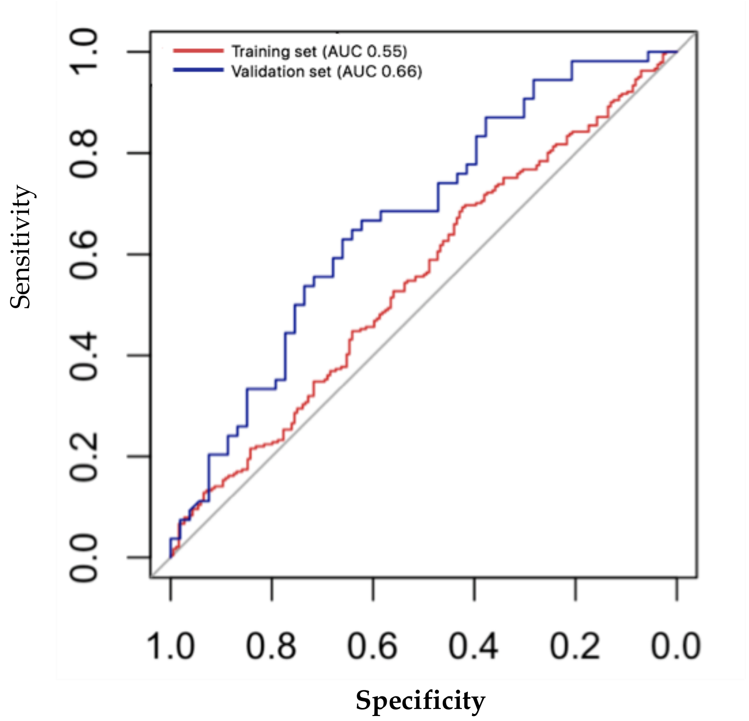

Figure S3. ROC-curve, which shows the accuracy of the linear discriminant model based on principal component reduction in the asthmatic population(training and validation set), to distinguish ever (n = 237) and never smokers (n = 295). Training set Case = 184; Control = 241: AUC: 0.55; 95% CI: 0.49–0.60; Validation set Case = 53; Control = 54: AUC 0.66; 95% CI 0.56–0.77.

Due to the extremely small proportion of non-smokers in the COPD group (number of ever-smokers = 356; never smokers = 8), we did not perform the same analysis in the COPD group of patients, considering that the accuracy of the model would not have been a good performance measure in this case.

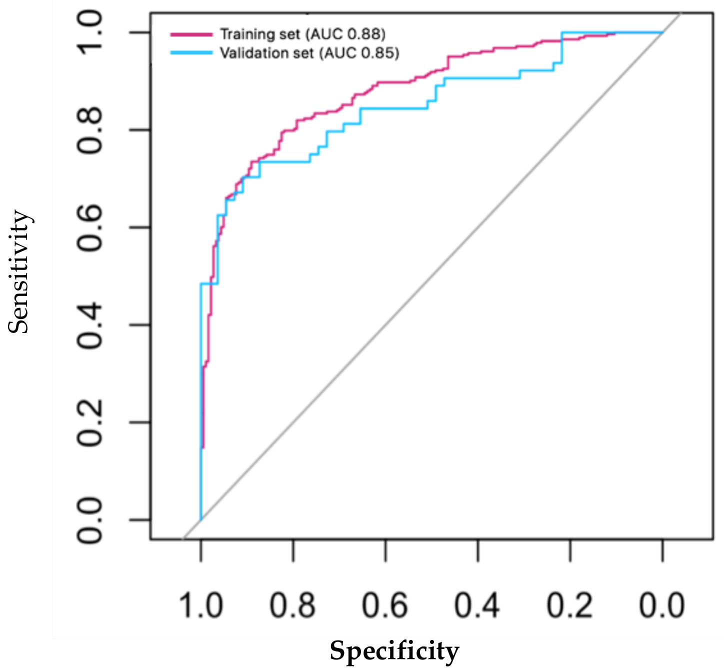

Figure S4. ROC-curve, which shows the accuracy of the linear discriminant model based on principal component reduction in the ever smokers population (train and validation set), to distinguish asthma (n = 237) and COPD (n = 356) patients. Training set Case = 182; Control = 292: AUC: 0.88; 95% CI: 0.85–0.91; Validation set Case = 55; Control = 64: AUC: 0.85; 95% CI: 0.78–0.92. .

Table S1. Demographics of asthma patients stratified by smoking status. Data are expressed in number of patients, mean ± standard deviation or median and range for non-normal distributions.

**Asthma patients**	**Ever-smokers (n = 237)**	**Never Smokers (n = 295)**	***p*-value**
Age (mean (SD))	56.22 (12.47)	47.91 (17.94)	<0.001
BMI (mean (SD))	28.92 (5.82)	26.88 (6.27)	0.002
Gender = M/F (%)	108/129 (45.6/54.4)	109/186 (36.9/63.1)	0.074
Allergy = Y/N (%)	121/59 (51.0/24.9)	207/88 (70.2/29.8)	0.803
FEV1 (mean (SD)) (l)	2.60 (0.80)	2.65 (0.92)	0.194
FEV1/VC (mean (SD)) (%)	66 (13)	70 (14)	0.007
Pack/year (median [IQR])	12.00 [4.50, 21.00]	0.00 [0.00, 0.00]	<0.001
ACQ (median [IQR])	1.57 [0.71, 2.29]	1.43 [0.71, 2.29]	0.037
ICS = N/Y (%)	31/206 (13.1/87)	46/249 (15.6/84.4)	0.034
Oral corticosteroids (%)			0.020

Yes, now	5 (2.1)	5 (1.7)	
Yes, before	34 (14.3)	32 (10.8)	
No	198 (83.5)	258 (87.5)	

Table S2. Demographics of COPD patients stratified by smoking status. Data are expressed in number of patients, mean ± standard deviation or median and range for non-normal distributions.

**COPD patients**	**Ever Smokers (n = 356)**	**Never Smokers (n = 8)**	***p*-value**
Age (mean (SD))	67.78 (8.93)	66.88 (10.51)	<0.001
BMI (mean (SD))	27.79 (5.74)	26.15 (5.84)	0.011
Gender = M/F (%)	175/181 (49.1/50.8)	4/4 (50.0/50.0)	0.508
FEV1 (mean (SD)) (l)	1.60 (0.67)	1.72 (0.85)	0.603
FEV1/VC (mean (SD)) (%)	60.97 (17.11)	71.88 (21.01)	0.161
Pack/year (median [IQR])	38.00 [20.25, 50.00]	0.00 [0.00, 0.00]	<0.001
CCQ (median [IQR])	2.00 [1.20, 2.80]	2.90 [1.68, 3.30]	0.231
ICS = No/Yes (%)	139/217 (39.0/60.9)	1/7 (12.5/87.5)	0.336
Oral corticosteroids (%)			0.830
Yes, now	9 (2.5)	0 (0.0)	
Yes, before	27 (7.6)	0 (0.0)	
No	320 (89.8)	8 (100.0)	
